# Integrated Jingmenvirus Polymerase Gene in *Ixodes ricinus* Genome

**DOI:** 10.3390/v14091908

**Published:** 2022-08-29

**Authors:** Evgeny S. Morozkin, Marat T. Makenov, Olga B. Zhurenkova, Ivan S. Kholodilov, Oxana A. Belova, Ekaterina V. Radyuk, Marina V. Fyodorova, Yana E. Grigoreva, Alexander G. Litov, Anna V. Valdokhina, Victoria P. Bulanenko, Andrei E. Samoilov, Elena V. Korneenko, Yana A. Voizekhovskaya, Alexey D. Neverov, Galina G. Karganova, Lyudmila S. Karan

**Affiliations:** 1 Group of Molecular Diagnostics and Epidemiology of Zoonotic Diseases, Department of Molecular Diagnostics and Epidemiology, Central Research Institute of Epidemiology, 111123 Moscow, Russia; 2 Laboratory of Biology of Arboviruses, Chumakov Federal Scientific Center for Research and Development of Immune-and-Biological Products of RAS (Institute of Poliomyelitis), 108819 Moscow, Russia; 3 Laboratory of Mathematical Biology and Bioinformatics, Research Institute for Systems Biology and Medicine, 117246 Moscow, Russia; 4 Multiomics Research Laboratory, Research Institute for Systems Biology and Medicine, 117246 Moscow, Russia; 5 Department of Virology, Faculty of Biology, Lomonosov Moscow State University, 119234 Moscow, Russia

**Keywords:** jingmenviruses, Alongshan virus, endogenous viral elements, Ixodes, ixodid ticks, tick cell line

## Abstract

Members of the jingmenviruses group have been found in arthropods and mammals on all continents except Australia and Antarctica. Two viruses of this group were isolated from patients with fever after a tick bite. Using a nested RT-PCR assay targeting a jingmenvirus polymerase gene fragment, we screened ticks collected in seven regions of Russia and found that the abundant jingmenvirus-positive were of *Ixodes ricinus* species, with the prevalence ranging from 19.8% to 34.3%. In all cases, DNase/RNase treatment suggested that the detected molecule was DNA and subsequent next generation sequencing (NGS) proved that the viral polymerase gene was integrated in the *I. ricinus* genome. The copy number of the integrated polymerase gene was quantified by qPCR relative to the ITS2 gene and estimated as 1.32 copies per cell. At least three different genetic variants of the integrated polymerase gene were found in the territory of Russia. Phylogenetic analysis of the integrated jingmenvirus polymerase gene showed the highest similarity with the sequence of the correspondent gene obtained in Serbia from *I. ricinus*.

## 1. Introduction

Segmented flavi-like viruses of the jingmenviruses (JMV) group were first discovered in 2014 in ixodid ticks in China [1]. Currently, this group refers to related, unclassified *Flaviviruses* and includes the Jingmen tick virus [1], the Alongshan virus [2], the Yanggou tick virus [3], the Takachi virus [4], and others. The Alongshan virus and the Jingmen tick virus were detected in patients with a febrile illness [2,5,6,7].

The viruses of this group have a genome segmented into four segments [1]. Segment 1 encodes a nonstructural protein (NSP1), which has homology to the flavivirus NS5 protein (RNA-dependent RNA polymerase). Segment 3 encodes a nonstructural protein that resembled the NS2b–NS3 complex of flaviviruses. The other two segments encode non-flavivirus-related proteins and appear to be unique for this virus group.

Representatives of JMV group were found in a number of ixodid tick species from several genera including *Rhipicephalus, Haemaphysalis, Amblyomma, Ixodes,* and *Dermacentor* [1,2,3,4,5,8,9]. JMVs were detected in China, Brazil, different parts of Europe, USA, Guinea, Turkey, Japan, Uganda, Lao People’s Democratic Republic, Cambodia, and Trinidad and Tobago [4,7,9,10,11,12,13,14,15,16]. In Russia, the Alongshan virus and the Yanggou tick virus were detected in *Ixodes persulcatus, Ixodes ricinus, Dermacentor reticulatus*, *Dermacentor marginatus*, *Dermacentor nuttalli,* and *Haemaphysalis concinna* ticks in different regions [3,8]. Furthermore, RNA of the Jingmen tick virus was present in the sera of patients with Crimean–Congo hemorrhagic fever in the Rostov region of Russia [6].

In the first publication describing the Jingmen tick virus, the authors only found evidence of the RNA form of the virus [1]. Similarly, when Maruyama et al. [17] discovered the Mogiana tick virus in Brazil they checked for the presence of viral DNA, but found only viral RNA. Almost all subsequent studies of JMV were performed as a viral RNA search, with no attention to viral DNA as no evidence was indicating its significance. There are two exceptions: the study describing the Takachi virus [4], where authors had confirmed the absence of DNA forms of the virus, and the work on RNA viruses of ixodid ticks in the French Antilles [18]. The authors of this work showed the presence of JMV-related DNA fragments in *Rhipicephalus microplus* from Guadeloupe and Martinique. All four segments of the Jingmen tick virus were found as DNA in tick samples [18]. However, the authors concluded that the detection of the viral DNA may be the result of residual RNA contamination [18].

Mostly, the presence of DNA sequences of non-retroviral RNA viruses in genomes of eukaryotic cells arise after the viral infection of a host’s germline cells and the integration of viral genes or genomes into a host’s chromosomes [19,20]. The term endogenous viral elements (EVEs) is usually used for integrated viral sequences in the animal genome. A number of non-retroviral EVE sequences belonging to several virus families were found in various animal genomes [19,20]. The search for EVEs is carried out mainly by bioinformatics/computational methods. Therefore, in regards to arthropods, the largest number of EVEs were found in *Aedes, Culex*, and *Anopheles* mosquitoes, since their genomes are better annotated [21,22,23,24,25,26]. Mosquito genomes most often contained EVEs of negative-sense RNA viruses (*Rhabdoviridae*, unassigned mononega-like viruses) [27]. Complete genomes of the hard ticks (Ixodidae) have only been annotated for a few species, and as a result, there are only a few works that have investigated EVEs in ticks. A number of EVEs were found in the genomes of *Ixodes scapularis* and *I. ricinus*; and phylogenetic analysis revealed that most of them are negative-sense RNA virus-derived sequences [27,28].

In this paper, we report that the JMV polymerase gene has been integrated into the *I. ricinus* genome.

## 2. Materials and Methods

### 2.1. Study Design

**Figure 1:**
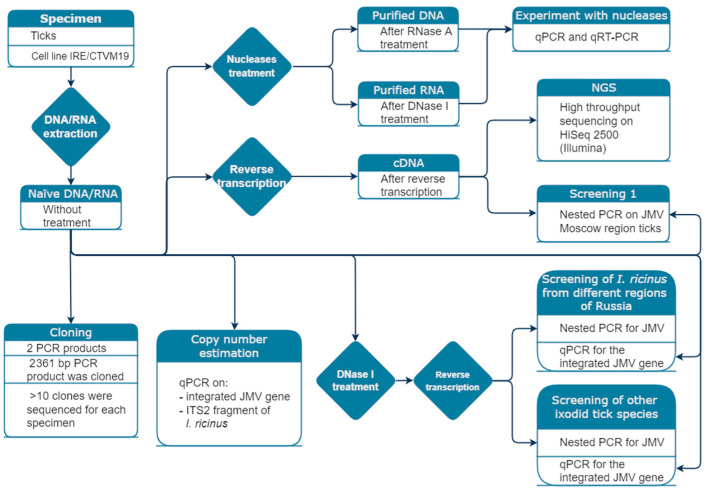
Study design diagram. The flow chart demonstrates different types of treatment and analysis performed to investigate the presence of JMV polymerase gene in ixodid ticks.

This study began from a tick survey in the Moscow region. Using system targeting of the JMV polymerase gene, we detected a high prevalence of RT-PCR-positive ticks. This led us to the hypothesis that the DNA form of the virus was integrated into the tick genome or the genome of a tick’s endosymbiont. To test this hypothesis, we performed DNase and RNase treatment and re-tested all samples for viral DNA (without a reverse transcription step) (Figure 1). Furthermore, we conducted high-throughput sequencing of several JMV-positive tick samples on HiSeq Illumina to illustrate the presence of sequences mapping to both the viral JMV polymerase gene and tick DNA. Subsequently, we also expanded the study by analyzing ixodid ticks collected in different regions of Russia for the presence of JMV DNA and/or RNA. To avoid confusion, we have created a flowchart that demonstrated the experimental strategy used in the study (Figure 1).

### 2.2. The Study Areas

**Figure 2:**
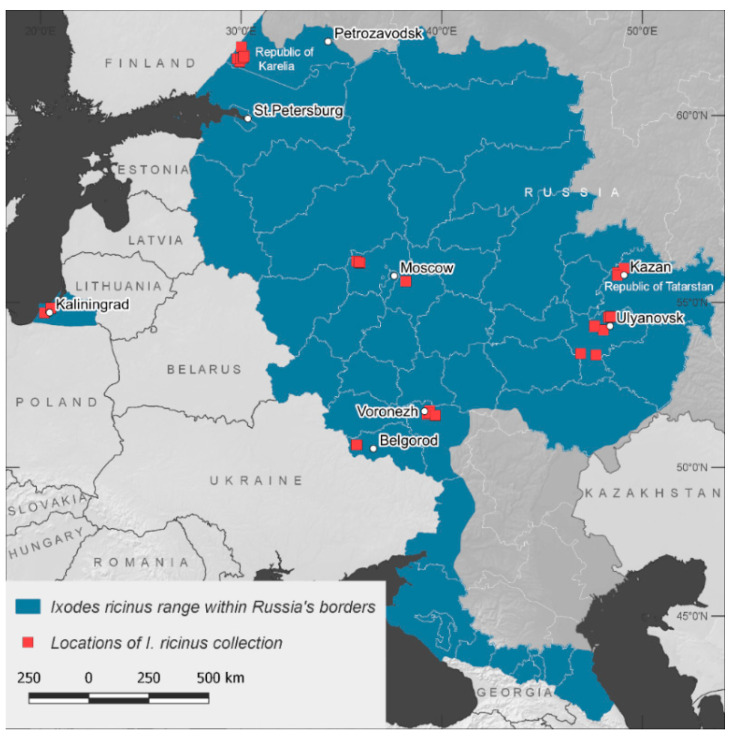
European part of Russia with depicted *Ixodes ricinus* collection sites.

The list of species included *I. ricinus*, *I. persulcatus*, *Ixodes trianguliceps*, *D. reticulatus*, *D. marginatus*, *D. nuttalli*, *H. concinna*, and *Haemaphysalis japonica*, which were collected in seven regions of Russia (Figure 2). More detailed information including the number of collected ticks and georeferenced data are presented in Table S1. Additionally, we used a collection of ticks published earlier [3].

### 2.3. Tick Sampling and Processing

Ticks were collected by flagging on transects and tick developmental stage, sex, and species were identified using morphological characteristics [29,30]. Additionally, species identification was confirmed by sequencing the *COI* gene fragment using primers described previously [31].

The ticks were handled in two ways: approximately half of the samples from the Moscow region (325 from 676 ticks) were processed individually (single tick per tube). For the remaining half (351 from 676), we pooled the ticks as follows: three adult ticks per pool for *D. reticulatus* and five adult ticks per pool for *I. ricinus*. Nymphs were studied separately (without pooling). *I. ricinus* ticks from other regions of Russia were tested in pools, while the other ixodid species were tested separately (without pooling). The ticks were washed in 70% alcohol and twice in 0.15 M NaCl, and then homogenized with TissueLyser LT (Qiagen, Hilden, Germany) in the following volumes of 0.15 M NaCl solution: 500 µL for *Dermacentor* ticks, 300 µL for adult ticks of *Ixodes* and *Haemaphysalis* genera, and 200 µL for *I. ricinus* nymphs. Pools were homogenized in 500 µL of 0.15 M NaCl.

Total nucleic acids were extracted from 100 μL of tick suspension using RIBO-prep kit (AmpliSens, Moscow, Russia) following the manufacturer’s instructions. Extracted nucleic acids were split into two aliquots that were treated with DNase I (Thermo Fisher Scientific, Vilnius, Lithuania) and Rnase A (Thermo Fisher Scientific, Vilnius, Lithuania), respectively. We used EcoRI endonuclease (Thermo Fisher Scientific, Vilnius, Lithuania) to distinguish between single- or double-stranded DNA. The nuclease treatment was carried out according to the manufacturer’s instructions. Purified RNA was DNase I treated and used to check for the presence of viral RNA.

### 2.4. PCR Assays

For PCR screening, JMV RNA was reverse transcribed using Reverta-L kit (Central Research Institute of Epidemiology, Moscow, Russia) following the manufacturer’s instructions. We developed nested PCR assays for the JMV polymerase gene to enable amplification of a 425-bp fragment in the first PCR round and a 233-bp fragment in the second PCR round. The reaction mix for each round contained a set of primers designed to amplify all related JMV, available in GenBank on April 2020 (Table 1). The detailed description of PCR conditions and reaction mix compositions is provided in Table S2. An aliquot of PCR product (5 μL) was run on a 3% agarose gel and visualized with UV illumination after staining with 0.5 μg/mL ethidium bromide.

Furthermore, we designed primers (Mos-Seq-F-2380 and Mos-Seq-R-2925) for amplification and sequencing of a 509 bp fragment in the 3′-end of the JMV polymerase gene for phylogenetic analysis (Table 1). The PCR was conducted with the conditions detailed in Table S2.

### 2.5. Quantitative PCR

To quantify the copy number of the integrated JMV polymerase gene, three qPCR assays were developed (Table 2) targeting the JMV polymerase gene, the genome integration site (a fragment of *I. ricinus* genome where the JMV polymerase gene was inserted), and the internal transcribed spacer 2 (ITS2) fragment of *I. ricinus*. Estimated limits of detection for the qPCR assays were approximately 1000 copies per ml. All reactions were performed in duplicates. Quantitative PCR amplification was performed with RotorGene Q instrument (Qiagen, Hilden, Germany). Reaction mix compositions and PCR conditions are detailed in Table S2. Tick-borne encephalitis virus (TBEV) RNA was detected using a commercial multiplex PCR kit (AmpliSens TBEV, *B. burgdorferi* s.l., *A. phagocytophilum*, *E. chaffeensis/E. muris*-FL; Central Research Institute of Epidemiology, Moscow, Russia) according to the manufacturer’s instructions. Tick-borne encephalitis virus strain Absettarov were obtained from Chumakov Institute of Poliomyelitis and Viral Encephalitides collection.

There is no information on the number of *ITS2* copies per genome of *I. ricinus*. However, such data exist for the closely related *I. scapularis*. According to NCBI, the genome of *I. scapularis* contains 442 copies of the *ITS2* region (Genome assembly ASM1692078v2), hence one diploid tick cell contains 884 copies of the *ITS2* region. We used this number as a divisor to calculate tick cells number in each sample:

$$N_{tick\;cells}=\frac{Copy\;number_{ITS2}}{2\times 442}.$$

Further, we used the copy number of the integrated JMV polymerase gene and the estimated number of tick cells to calculate the copy number of the integrated JMV polymerase gene per cell:

$$Copy\;number_{insertion\;per\;cell}=\frac{Copy\;number_{insertion\;per\;sample}}{N_{tick\;cells}}$$

### 2.6. Sequencing and Cloning

#### 2.6.1. Next Generation Sequencing

A total of six JMV-positive ticks collected in the Moscow region were used for the library preparation. Naïve RNA/DNA templates without any nuclease treatment were reverse-transcribed using Reverta-L kit (Central Research Institute of Epidemiology, Moscow, Russia). Then, 20 μL of first strand cDNA product was immediately taken into second strand cDNA synthesis using NEBNext^®^ Ultra™ II Non-Directional RNA Second Strand Synthesis Module, which is a part of NEBNext^®^ Ultra II RNA Library Prep Kit for Illumina (New England BioLabs, Ipswich, MA, USA). DNA fragmentation was performed in the Focused-ultrasonicator ME220 (Covaris, Woburn, MA, USA) according to the manufacturer’s instructions with parameters for obtaining 500-bp fragments. The remaining steps of library preparation were completed using the NEBNext Ultra II DNA Library Prep Kit for Illumina (New England BioLabs, Ipswich, MA, USA). Size selection of the final libraries to ~400–500 bp library fragment size was performed using SPRIselect (Beckman Coulter, Danvers, MA, USA) solid-phase reversible immobilization beads. The quality and fragment length distribution of the obtained libraries were evaluated with Agilent Bioanalyzer 2100 (Agilent Technologies, Waldbronn, Germany). Sequencing was performed with HiSeq PE Rapid Cluster Kit v2 and HiSeq Rapid SBS Kit v2 (500 cycles) on HiSeq 2500 (Illumina, San Diego, CA, USA).

#### 2.6.2. Sanger Sequencing

Purified PCR products were sequenced bidirectionally using the BigDye Terminator v1.1 Cycle Sequencing kit (Thermo Fisher Scientific, Austin, TX, USA) on an Applied Biosystems 3500xL Genetic Analyzer (Applied Biosystems, Foster City, CA, USA).

#### 2.6.3. Cloning

We obtained a long PCR product of 2361 bp for two JMV-positive samples using the MS-F-875 (5′-TCGGATAGGCTGGAGACTCA-3′) and MS-R-3100 (5′-TGACGAGATGCCGGTTCCCGA-3′) primers. These primers flank the overlapping region of JMV polymerase gene and an *Ixodes*’ genomic sequence. More detailed data on reaction mix composition and PCR conditions are provided in Table S2. PCR product (5 μL) was run on a 1.5% agarose gel and visualized using UV illumination after staining with 0.5 μg/mL ethidium bromide. The fragment was directly ligated and cloned in pGEM-T vector (Promega, Madison, WI, USA). Positive clones were screened by PCR with primers flanking the vector and inserts and verified by Sanger sequencing using three pairs of primers (Table S3). For each sample, at least 10 clones were selected for sequencing.

### 2.7. Data Analysis

The 95% confidence intervals of the prevalence for the unpooled ticks were estimated using exact Clopper–Pearson methods in R in the package ‘PropCIs’ [33]. Pooled tick prevalence for the fixed pool size and confidence limits were estimated using a Bayesian approach and a Gibbs sampler [34,35]. Alpha and beta values for the prior distributions of true prevalence and test sensitivity and specificity were calculated based on unpooled data. The simulation was run on the EPITOOLS web platform for 25,000 iterations, with 5000 iterations discarded to allow for convergence [35]. For a part of the analyzed ticks, pooled into pools of variable size (1 to 23 ticks per pool), we calculated the prevalence using maximum likelihood estimator with the assumption of 100% test sensitivity and specificity [36]. The calculations were performed on the EPITOOLS web platform (https://epitools.ausvet.com.au, accessed on 26 August 2022).

The nucleotide sequences obtained by sequencing were aligned, compared, and analyzed using ClustalW and BLAST. Gblocks (version 0.91b, Castresana J., Heidelberg, Germany) was used to remove divergent or ambiguously aligned regions [37]. Mega X package [38] was used for phylogenetic analyses. Phylogenetic trees were constructed using the maximum-likelihood method with the general time-reversible model [39] and the Tamura 3-parameter model [40], with bootstrap analysis based on 1000 replicates.

Raw reads of high-throughput sequencing were filtered with Trimmomatic [41] using the SLIDINGWINDOW:4:20 MINLEN:40 parameters. Genome assemblies were completed by using SPAdes 3.14 [42] with default parameters. *Flaviviridae* sequences were selected by calling BLASTn [43] on assembled contigs using all of the available *Flaviviridae* nucleotide sequences available in the nucleotide database.

## 3. Results

### 3.1. High Prevalence of Jingmenviruses Positive Ticks in Moscow Region

A total of 676 ticks were collected in the Moscow region, 325 of them were analyzed individually, and 351 ticks were pooled (Table 3). Total nucleic acids extracted from ticks and tick pools were reverse transcribed and screened for the JMV fragment with the nested PCR assay. We found 66 PCR-positive specimens (Table 3), 49 of which were confirmed by sequencing with the nested PCR primers (see Table 1, ‘Multiplex Mix In’), and/or Mos-Seq-F-2380, Mos-Seq-R-2925 (Table 1). The remaining 17 positive specimens were confirmed with qPCR assay for the integrated JMV polymerase gene (Table 2).

The prevalence of JMV-positive *I. ricinus* ticks was 35.1% (CI95%: 27.6–43.2%) for unpooled samples and 27.4% (CI95%: 21.0–34.7%) for pooled samples (Table 3). All samples of *D. reticulatus* yielded negative results in the PCR assay. JMV cDNA was found in adult *I. ricinus* ticks (both females and males) as well as in questing nymphs and larvae. The sequencing of positive samples with the JMTV-In-F/JMTV-In-Rev primers (Table 1) confirmed their JMV group origin and demonstrated high similarity to the nucleotide sequence of Serbia-24-28 (MT822179) obtained from *I. ricinus* in Serbia [44].

### 3.2. RNA Virus or DNA Form?

To test the hypothesis that we detected DNA fragments derived from JMV and not the viral RNA, we used 10 JMV-positive ticks and the TBEV strain Absettarov as a positive control of the RNA virus. Total nucleic acids were treated with either DNase I or Rnase A and then tested by qPCR or qRT-PCR using the primers IRJPG-NS5–F, IRJPG-NS5–Rev, IRJPG-NS5-Probe (Table 2), and commercial kit for TBEV detection. Expectedly, the TBEV-positive control yielded positive signals in the qRT-PCR for both naïve RNA/DNA and DNase I-treated, but not RNase-treated samples (Table 4). In comparison, for JMV-positive ticks the results were quite different. All 10 tested specimens were negative for viral RNA (after DNAase I treatment), thus revealing that all 10 tested JMV-positive ticks contained the DNA form of the JMV polymerase gene.

Encouraged by the results, we re-analyzed all ticks collected in the Moscow region looking for viral DNA by the nested PCR assay without reverse transcription. DNA fragments of the JMV polymerase gene were detected in all previously JMV-positive specimens. Additionally, the treatment of samples with EcoRI resulted in signal loss in the PCR assay indicating that the detected JMV polymerase gene fragment is double-stranded DNA.

### 3.3. Is the DNA Fragment Integrated in the Tick Genome?

#### 3.3.1. NGS and Further Confirmation by Sanger Sequencing

**Figure 3:**
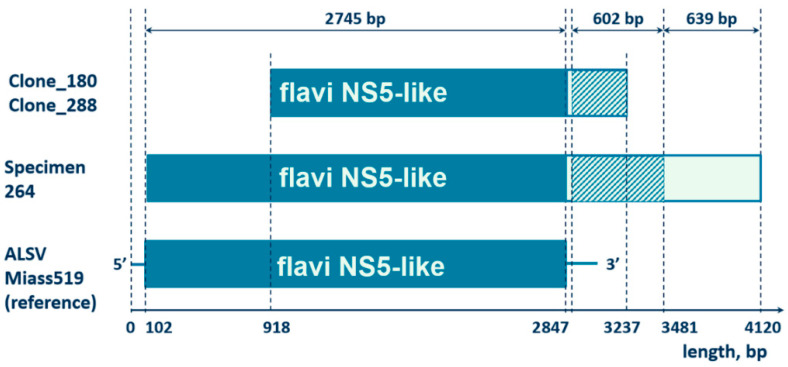
Graphical alignment of a 4005 bp contig of a JMV-positive sample obtained by HiSeq (specimen 264), two cloned samples (id 180 and 288), and a reference sample-segment 1 of Alongshan virus, strain Miass519 (GenBank accession number MN648774). The dark blue boxes represent the coding sequence of the jingmenvirus polymerase gene, the hatched box corresponds to the fragment of the *Ixodes scapularis* genome (GenBank accession number XM_042293990). The light blue box represents the fragment with an unknown sequence. The horizontal axis shows the length of the nucleotide sequences.

We have performed high-throughput sequencing of cDNA from six samples of JMV-positive *I. ricinus* ticks. *De novo* genomic assemblies of all these samples contained contigs of the JMV polymerase gene, and one sample yielded a contig in which a large fragment of the coding region of the JMV polymerase gene (2733 bp) was connected downstream to an unknown sequence of 1273 bp (Figure 3, Specimen 264). The BLASTx algorithm comparing translated nucleotide sequences showed similarity of the unknown sequence to an uncharacterized *I. scapularis* protein LOC115330668 (GenBank accession number XP_042148831, 62% identity) and a hypothetical protein HPB47_003995 from *I. persulcatus* (GenBank accession number KAG0419594, 58% identity) (Figure 3). The other 639 bp of the unknown sequence did not map to any sequences in GenBank by either BLASTn or BLASTx algorithms.

Subsequently, the overlapping of the JMV polymerase gene and the unknown sequence was confirmed by PCR and Sanger sequencing using primers flanking the 3′-end of the JMV polymerase gene and the *Ixodes’* gene (Table S3, MF2835 and MR3100). Ten positive ticks were sequenced, confirming the overlapping (Table S4). Furthermore, we obtained 2361 bp amplicons that cover a fragment of the JMV polymerase gene (1950 bp) and the unknown sequence on the 3′-end (411 bp) and cloned them in the pGEM-T vector (see primers MS-F-875 and MS-R-3100 in the Methods section). Sequencing of the clones also confirmed the overlapping of the JMV polymerase gene and an *Ixodes’* gene (Table S4).

Both *I. scapularis* and *I. persulcatus* ticks belong to the subgenus *Ixodes* Latreille, 1795, which is also true for *I. ricinus* [29,45]. Therefore, presence of a hypothetical protein sequence from these two *Ixodes* species allows us to assume that the genome of *I. ricinus* also contains the same or a very similar gene. To verify this, we designed a qPCR assay for the detection of the integration site in the *I. ricinus* genome (Table 2, primers Ir-3100-F, Ir-3100-R, and Ir-3100-probe) and used it to screen the *I. ricinus* collection. As a result, we found the integration site in all tested specimens of *I. ricinus*, both JMV-positive and JMV-negative. Furthermore, we found the integrated JMV polymerase gene in the uninfected *I. ricinus* cell line IRE/CTVM19 [46]. Thus, we confirmed our hypothesis that the integration site is a part of the tick genome, and the DNA form of the JMV polymerase gene is inserted into the *I. ricinus* genome. Interestingly, no other integrated segments of JMV were not found after the analysis of high-throughput sequencing data.

#### 3.3.2. Quantifying the Integrated Jingmenvirus Polymerase Gene in Ixodes Ricinus Genome

**Figure 4:**
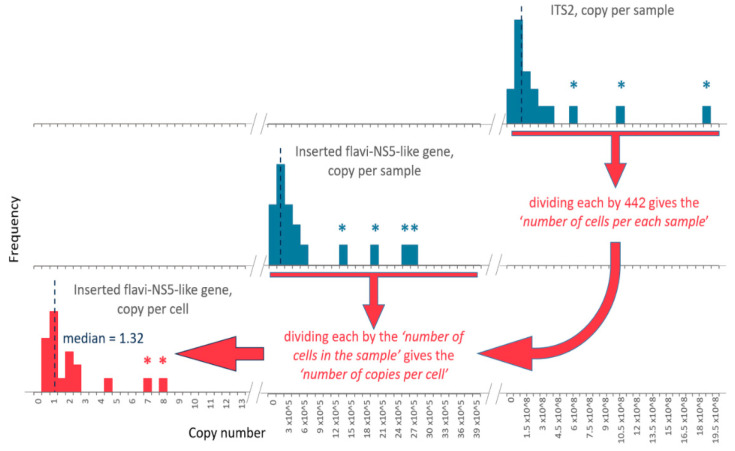
The jingmenvirus polymerase gene copy number estimated by two qPCR assays. Histograms represent the distribution of the obtained copy number values. ITS2 is the internal transcribed spacer of the *I. ricinus* nuclear genome. Dashed vertical lines show median for each plot. Outliers shown by asterisks were excluded from calculating the median.

We developed a qPCR assay to estimate the copy number of the integrated JMV polymerase gene relative to the *I. ricinus* gene with a known copy number (Figure 4). Currently, the genome of *I. ricinus* has not been annotated. At the same time, there is a datum on the number of nuclear ribosomal genes for a closely related *I. scapularis*—(442 copies per genome). Therefore, we decided to use the ITS2 fragment as a reference gene in this assay. A total of 18 *I. ricinus* ticks were individually tested by qPCR (as naïve DNA without reverse transcription) including eight nymphs, five females, and five males. Two samples with the highest values of the copy number were excluded from consideration as outliers exceeding the interquartile range by 1.5-fold (Table S5). As a result, the median copy number of the integrated JMV polymerase gene was calculated to be 1.32 copies per cell (Table S5).

### 3.4. Screening for Jingmenviruses and the Integrated Jingmenvirus Polymerase Gene in Ixodes Ricinus from Different Regions of Russia

A total of 140 pools of *I. ricinus* (451 ticks) from six regions were tested using two qPCR assays: (1) qPCR specific for the integrated JMV polymerase gene (Table 2); and (2) nested PCR using cDNA as a template, which was obtained after DNase I treatment of naïve RNA/DNA (Table 5). The collection sites of these ticks are widely distributed across the Russian part of the *I. ricinus* geographic range, covering the northern, eastern, and southern parts of the global range of the species (Figure 2). DNA fragments of the JMV polymerase gene were detected in samples from each region. Due to the pool size variability of the samples (from 1 to 10 for adult ticks and from 5 to 23 for nymphs), we estimated the prevalence of ticks with the integrated JMV polymerase gene using the maximum likelihood method, assuming 100% test sensitivity and specificity. The observed prevalence varied from 10.1% to 36.7% (Table 5). Two screened samples were PCR-positive for the Alongshan virus and sequencing revealed the presence in these samples of both Alongshan virus RNA and DNA of the integrated jingmenvirus polymerase gene. However, it is important to note, that both Alongshan-positive samples were pools of five ticks per sample. Therefore, we cannot conclude that the Alongshan virus was found in the same tick that also had the integrated JMV polymerase gene.

### 3.5. Screening for Jingmenviruses and the Integrated Jingmenvirus Polymerase Gene in Other Ixodid Tick Species

A total of 412 ticks representing eight species collected in the different regions of Russia were analyzed by qPCR specific for the integrated JMV polymerase gene (Table 2) and nested PCR on cDNA obtained after the DNase I treatment of naïve RNA/DNA (Table S6). This screening revealed five specimens positive for the Alongshan virus and two specimens positive for the Yanggou tick virus. These findings were described in our previous paper [3]. Here, we analyzed the samples for the presence of the DNA fragments of the integrated JMV polymerase gene, which were not detected in any of the ticks (Table S6).

### 3.6. Genetic Variability of the Jingmenvirus Polymerase Gene Fragment in the Studied Ticks

**Figure 5:**
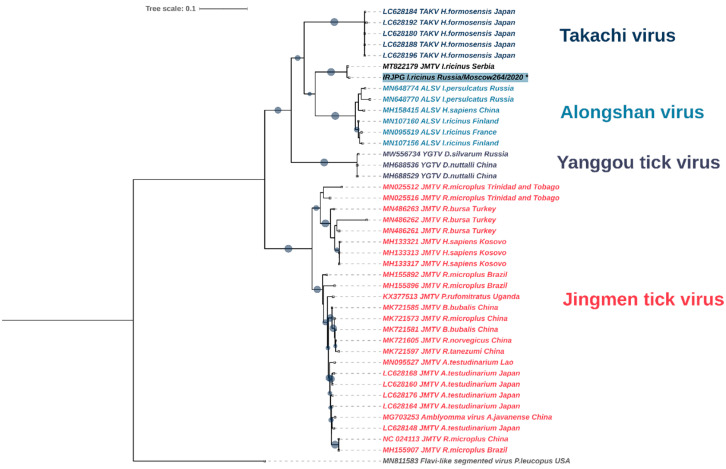
Phylogenetic tree of the integrated jingmenvirus polymerase gene (826 aa fragment) in comparison with jingmenviruses. The phylogenetic analyses were inferred by using the maximum likelihood method with 1000 pseudoreplicates. The Akaike information criterion was chosen as a model selection framework and the general time-reversible model as the best model. Maximum likelihood method bootstrap replicates (≥70%) are indicated. The sequences from the Moscow region generated during this study are indicated with an asterisk and blue filled text background. Scale bar indicates the mean number of nucleotide substitutions per site. The filled circles on branches indicate the bootstrap value greater than 0.9.

Phylogenetic analysis of the JMV polymerase gene based on the 2730 bp fragments showed that the discovered integrated DNA (*I. ricinus* Russia Moscow-264 isolate) is the most similar (*p*-distance = 0.012) to the sequence of the JMV polymerase gene obtained in Serbia from *I. ricinus* (GenBank accession number MT822179, Figure 5). Additionally, GenBank contains a similar sequence (GenBank accession number JXMZ02142294, Figure 5), which was annotated in the genomic DNA of the *I. ricinus* strain (Charles River) obtained by whole genome shotgun sequencing (*p*-distance = 0056). As a result, the integrated JMV polymerase sequence together with the sequences obtained from Serbian ticks form a separate cluster in the JMV group and shows a close relationship to the JMV polymerase gene of the Alongshan virus (Figure 5).

**Figure 6:**
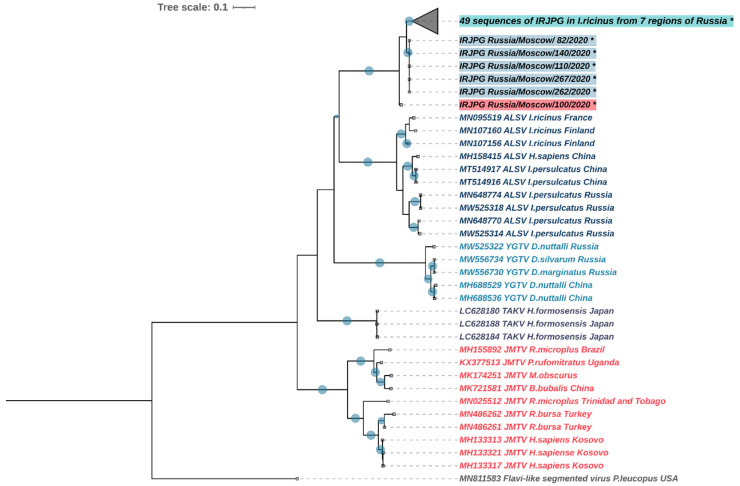
Phylogenetic analysis of the integrated jingmenvirus polymerase gene based on a 509 bp fragment. The phylogenetic analyses were inferred by using the maximum likelihood method with 1000 pseudoreplicates. The Akaike information criterion was chosen as a model selection framework and the Tamura-Nei model as the best model. Maximum likelihood method bootstrap replicates (≥70%) are indicated by filled circles. The sequences obtained in this study are indicated with asterisks and filled text background. Scale bar indicates the mean number of nucleotide substitutions per site.

The genetic variability of the integrated JMV polymerase gene in ticks is also noteworthy. Sequencing of the 3′ end region of the gene (509 bp fragment) from 35 ticks from the Moscow region and 24 ticks from other regions of Russia revealed the presence of at least three different genetic variants (Figure 6). The first genovariant, represented in our sample by one specimen collected in the Moscow region (id 100), appears to have been the earliest deviation into a separate branch and has a 23–29 nt difference to the other genovariants (*p*-distances—0.045–0.057). The second genovariant was present in seven samples and is removed from the others by a distance of 17–23 nt (*p*-distances—0.033–0.045). This genovariant was isolated from ticks collected in the Moscow, Kaliningrad, and Voronezh regions (Figure 2). Two of the samples simultaneously contained the first and the second genovariants. One of these samples was a pool of five ticks, and the second sample was an adult female of *I. ricinus.*

The third genovariant in the clade was the most abundant and included 51 sequences obtained from the Moscow region, the Republic of Karelia, the Republic of Tatarstan, Voronezh, Kaliningrad, Belgorod, and Ulyanovsk regions of Russia. Moreover, a sequence from GenBank obtained from *I. ricinus* in Serbia (accession number MT822179) also belongs to this cluster (Figure 6). Notably, the ticks from the Moscow region in all three clusters were collected on the same path and the distance between the collection sites did not exceed 1 km.

## 4. Discussion

In our study, we clearly demonstrate the integration of the JMV polymerase gene fragment into the genome of *I. ricinus* from seven regions of Russia. A number of other ixodid species inhabiting Russia were free from the integrated JMV polymerase gene, but additional studies of different tick species in an expanded geographic range are needed to validate a more general assumption. Based on the phylogenetic study of the hard ticks [47], the most promising strategy could be to look at the closely related species of the genus *Ixodes*: *I. acuminatus*, *I. redikorzevi*, *I. persulcatus,* and *I. pavlovskyi*.

The possible integration of viral sequences into the host genome was previously investigated by other authors working with viruses of the JMV group. For example, when the Jingmen tick virus was first discovered, the presence of DNA forms of this virus in the ticks’ samples was excluded based on the absence of an amplicon in PCR (without reverse transcription) of total DNA [1]. In the work reporting the first detection of the Mogiana tick virus-related nucleic acid sequences in *R. microplus* ticks in Brazil, the authors checked whether these sequences are of RNA or DNA origin [17]. A comparison of the amplification efficiency starting from either DNA or cDNA isolated from ticks showed that the virus-related sequences were in the form of RNA, and the DNA form was absent [17]. Discovering the Guaico Culex virus, Ladner et al. also interrogated the nature of the genomic material using nuclease treatment (DNase I and RNase A) and confirmed that all five genome segments were single-stranded, positive-sense RNA, and DNA forms were absent [48]. In the work describing the Takachi virus (JMV group), authors checked the presence of DNA forms by PCR without reverse transcription [4]. In subsequent works, only NGS sequencing of the virus was carried out and the presence/absence of DNA forms was not accounted for [2,7,9,10]. In these works, library preparation was done after DNase treatment, therefore, even if DNA forms were present in the samples, they would have been lysed with nucleases. The presence of DNA forms of JMVs was detected in *R. microplus* from the Antilles, and both viral RNA and DNA of all four segments of the viral genome were found in 1–8% of JMV-positive samples [18]. Because of this key difference, we cannot argue that the authors also found insertions into the tick genome. The authors themselves questioned the validity of their findings of the JMV DNA form and suggested contamination as a possible explanation.

On the other hand, in the study of ter Horst et al. [28], genomes of arthropods, including *I. ricinus* and *I. scapularis,* were screened for the presence of EVEs. In the genome of *I. scapularis* and *I. ricinus*, 387 and 168 viral sequences were found, respectively [28]. One of the sequences found in the *I. ricinus* genome (GenBank accession number: JXMZ02142294) corresponds to the polymerase gene of the Jingmen tick virus. Phylogenetic analysis showed that this sequence is closely related to the Serbian sequence MT822179 [44] and to the JMV polymerase gene inserted in the *I. ricinus* genome detected in our study (Figure 5). Importantly, the preparation of Serbian *I. ricinus* samples did not involve DNase treatment. Based on the genetic similarity of the Serbian sequences to the one we describe, the fact that they were both found in *I. ricinus*, and the presence of only the JMV polymerase gene and that no other viral genome segments were found, we can conclude that the sequences (GenBank accession numbers MT822179, MT822180) deposited by Stanojević et al. [44] come not to the Jingmen tick virus directly, as the authors pointed out, but are probably also an integrated DNA of the JMV polymerase gene.

Our phylogenetic analysis showed that the integrated fragment of the JMV polymerase gene is variable and forms at least three genovariants sufficiently removed from each other (range of *p*-distances 0.033–0.057). There are two possible explanations for the genetic heterogeneity of the insertion: (1) the integration event occurred once and the observed variability have been accumulated by the DNA form; (2) observed variability had existed in the ancestral virus population before the integration, and integration events occurred several times with the different genovariants. It is known that the mutation rate for virus-derived sequences integrated into a eukaryotic genome is lower than for the RNA viruses [49], so the observed genetic divergence had most likely been accumulated before integration into the *I. ricinus* genome. This suggests that insertion into the *I. ricinus* genome has occurred several times, and these insertion events happened in germline cells. One of the insertion’s genovariants was widespread in the *I. ricinus* area. Therefore, we can conclude that the insertion into the *I. ricinus* genome of this variant occurred long enough to spread within the species range. Our study showed that in the Moscow region the prevalence of ticks with this insertion was approximately 27–35%. The fact that not all studied *I. ricinus* had this insertion suggests that the insertion event has occurred after *I. ricinus* split into a separate species. Thus, the insertion of the JMV polymerase gene could potentially become a convenient marker for studying the genetic structure and phylogeography of *I. ricinus* populations.

While screening our *I. ricinus* collection, we found two specimens that were PCR-positive for both the JMV-integrated polymerase gene and the Alongshan virus. Both Alongshan-positive samples were pools of five ticks, preventing us from making a conclusion that the virus could successfully replicate in ticks with the integrated JMV polymerase gene. However, we also found the integrated gene in uninfected cell culture IRE/CTVM19. This cell culture has previously been used to successfully isolate the Alongshan and Yanggou tick viruses [3,8]. This suggests that the integrated JMV polymerase gene does not significantly affect the ability of the Alongshan virus and the Yanggou virus to replicate in tick cell culture. It is possible that virus-derived DNA of ancestral JMV could be involved in the antiviral immune response of host cells and lead to increased tolerance (low damage for host cell and high virus load). It was shown that during RNA-virus infection, *Aedes* mosquitos generate virus-derived DNA that is essential for viral tolerance [50]. A similar mechanism of tolerance to arboviruses could potentially exist in ticks even before the integration event.

We were unable to pinpoint the location of the insertion in the *I. ricinus* genome because the tick genome has not yet been annotated. The insertion was found in ticks of both sexes, which allows us to conclude that the JMV polymerase gene is not integrated into the sex Y chromosome. The assessment of the copy number of the insertion showed that one tick cell contains, on average, 1.5 copies of the insertion. Therefore, we hypothesize that only one copy of the JMV polymerase gene was inserted in the haploid genome of *I. ricinus*. Respectively, in a diploid genome, this number would be increased to two copies per cell and lower values do not contradict the single insertion hypothesis. Indeed, the insertion could be present in both the biallelic variant and in the single allelic variant (hemizygote) of the tick’s genome. A monoallelic variant is possible because not all individuals in the population have this insertion (particularly in the Moscow region population where the prevalence of ticks with insertion was around 27–35%). The offspring of two ticks, one with the insertion and one without, will inherit a monoallelic variant of the insertion. With that, the copy number estimation in our work has a certain limitation in that it is based on the data for the *I. scapularis*, not *I. ricinus*, and therefore this issue requires deeper investigation. Regarding the copy number of non-retroviral integrated RNA virus sequences, in *Aedes* mosquitoes, up to 71% of the virus-derived sequences had at least one additional copy in the mosquito’s genome [27]. In contrast, in the genome of *I. scapularis,* only 18% of all virus-derived sequences had an additional copy [27]. Our results about the copy number of the integrated fragment are thus consistent with what has been observed for *I. scapularis*.

Russo et al. [27] showed that among the non-retroviral integrated RNA virus sequences in *I. scapularis*, 81% is accounted for the genes encoding RNA-dependent RNA polymerase (RdRp gene), and the remaining 19% are from structural genes (nucleoprotein, glycoprotein, and matrix regions). In the case of *I. scapularis,* when the high frequency of RdRp integration was observed, the suggested explanation was an unusual template preference for the endogenous reverse transcriptases and integrases, which could facilitate the integration of virus-derived sequences in *I. scapularis* [27]. We cannot directly extrapolate the results of the study by Russo et al. [27] to our findings. However, we can speculate that for the endogenous reverse transcriptases there could also exist a template preference for the JMV polymerase gene of segmented positive-sense RNA viruses.

The presence of the reverse transcriptase enzyme is required to make a DNA copy from the viral RNA template. Presumably, non-retroviral integrated RNA virus sequences use the reverse transcriptase encoded by the retroelements abundant in eukaryotic genomes [19]. Indeed, some studies of EVE in insect genomes showed that retrotransposons are involved in reverse transcription and the integration of viral genomic fragments [50,51,52,53,54]. Particularly, for the *Aedes* mosquito genome, there is clear evidence for the association of RNA-virus-derived sequences and long-terminal repeat (LTR) retrotransposons [52,55,56]. For ixodid ticks, Russo et al. [27] suggested that the reverse transcriptase activity of non-long-terminal repeat (non-LTR) retrotransposons, particularly I, L1, and R1 elements, might be responsible for the appearance of RNA virus-derived sequences.

## 5. Conclusions

In conclusion, we would like to highlight the following main results of our work. Populations of *I. ricinus* ticks contain a JMV polymerase gene, which was integrated into the genome after separation of *I. ricinus* as a species. Insertion has occurred several times and long ago enough for ticks with the insertion to spread widely within the species range.

## Figures and Tables

**Table 1:** Oligonucleotide sequences of the primers used in PCR amplification and sequencing.

Name		Nucleotide Sequence (5′–3′)	Target	Length of the Product, bp
Nested PCR for screening on JMV				
Yanggou-OUT -F	Multiplex Mix Out	AAC GTG GAA AGG AAG AAT GGA	Jingmenvirus polymerase gene	425 (first round)
JMTV-OUT-F		AGA GAG GCA GAG AGG AAT GGA		
Alongshan-OUT-F		AAA GRG GGA AGG ARG AGT GGA		
Yanggou-OUT-R		TCT ATC CTT GCG TCT TTC TAC CA		
JMTV-OUT-R		TCT GTC SGC TCT YCG CTC CCG GA		
Alongshan-OUT-R		TCT GTC YTT CCT CCT CTC TGC CA		
JMTV-IN-F	Multiplex Mix In	GAG ACC TTC AAA AGR GAC CA	Jingmenvirus polymerase gene	~230 (second round)
Alongshan-IN-F		GAG GCC TTC AAG AGG GAC CA		
Alongshan-IN-Rev		TAC ATG ACC GTG TTG GAG AGG CGG T		
JMTV-IN-Rev		TAC ATC ACR GTG TTG GAC AGC CTG T		
Yanggou-IN-Rev		TAC ATA ACT GTG TTC GAG AGT CTT TC		
PCR for sequencing of 509 bp fragment				
Mos-Seq-F-2380		TGCTCCCATCAGTACTGGCCT	Jingmenvirus polymerase gene	509
Mos-Seq-R-2925		CGCCACCCCCGCAACCTGGT		

**Table 2:** Oligonucleotide sequences of the primers and probes used in qPCR assays.

Name	Nucleotide Sequence (5′–3′)	Target
qPCR assay for the integrated Jingmenvirus polymerase gene		
IRJPG-NS5 -F	GAGACCTTCAAAAGRGACCA	Jingmenvirus polymerase gene
IRJPG-NS5 -Rev	TACATCACRGTGTTGGACAGCCTGT	
IRJPG-NS5-Probe	ROX-TGGCAGGTGAAGACAAACAAGGG-BHQ2	
qPCR assay for the target site * detection		
Ir-3100-F	ACCGGCATCTCGTCAAGACGA	Integration site *
Ir-3100-R	AGATACTGTCTCTGCCACTCGT	
Ir-3100-probe	ROX-TCCCTCGACCCCAAGCATCGTGA-BHQ2	
qPCR kit for ITS2 fragment detection		
ITS2_B-TicksRP-F	KACRGAGTTCGTYGGCGCGT	ITS2
[32]		
ITS2_B-TicksRP-R	TGCAAATCAACGCCACGAGA	
[32]		
Probe-Ricin1	ROX-TTAATGGCGGACGCCGCGTTTCAAACGC-BHQ2	
[32]		

*—fragment of *I. ricinus* genome where the jingmenvirus polymerase gene was inserted.

**Table 3:** Prevalence of PCR-positive samples on Jingmenviruses in studied ticks of Moscow region.

Tick Species	Number of Ticks	Number of Formed Pools	Number of Ticks Per Pool	PCR-Positive Ticks on JMV Number–Prevalence, % (CI95%)
**Unpooled samples**				
*Ixodes ricinus*	154	154	1	54–35.1 (27.6–43.2)
*Dermacentor reticulatus*	171	171	1	0–0 (0.0%)
**Pooled samples**				
*Ixodes ricinus*	220	44	5	11–27.4 (21.0–34.7)
*Ixodes ricinus* Larvae	32	2	9 and 23	1 *
*Dermacentor reticulatus*	99	33	3	0–0 (0.0%)

*—one pool with 23 larvae of *I. ricinus* were positive in PCR; and we did not calculate prevalence for this case.

**Table 4:** The Ct-value of ten jingmenvirus-positive ticks and TBEV strain before and after processing with DNase I or RNase A in the qRT-PCR assay.

Sample’s ID	qRT-PCR		
	Naïve	After DNase I	After RNase A
*I. ricinus*-184	21.22	no signal	23.1
*I. ricinus*-723	21.94	no signal	23.91
*I. ricinus*-727	22.19	no signal	24.35
*I. ricinus*-746	21.27	no signal	23.65
*I. ricinus*-32654	21.47	no signal	23.68
*I. ricinus*-32659	26.78	no signal	29.30
*I. ricinus*-22684	19.16	no signal	21.37
*I. ricinus*-22687	29.68	no signal	31.72
*I. ricinus*-22689	27.73	no signal	29.94
*I. ricinus*-17433	22.54	no signal	24.43
TBEV strain Absettarov	20.92	22.92	no signal

**Table 5:** The number of the integrated jingmenvirus polymerase gene PCR-positive samples of *Ixodes ricinus* collected in other regions of Russia.

Region	Number of Ticks	Number of Formed Pools	Number of PCR-Positive on JMV Pools/Prevalence, % (CI95%) *
**Integrated jingmenvirus polymerase gene**			
Belgorod region	97	21	10/12.8 (6.6–17.6)
Republic of Tatarstan	70	10	9/19.5 (9.6–33.6)
Ulyanovsk region	49	14	10/36.7 (19.1–59.2)
Republic of Karelia	72	33	10/15.3 (8.0–25.3)
Voronezh region	62	16	10/25.4 (13.2–42.0)
Kaliningrad region	102	49	10/10,1 (5.2–17.0)
**Alongshan virus**			
Republic of Tatarstan	70	10	1/1.5 (0.0–6.6)
Ulyanovsk region	49	14	1/2.1 (0.1–9.1)

*—The prevalence was estimated for variable pool sizes with the assumption of 100% test sensitivity and specificity.

## Data Availability

Not applicable.

## Supplementary Materials

The following supporting information can be downloaded at: https://www.mdpi.com/article/10.3390/v14091908/s1, Table S1: Georeferenced data of collected ticks; Table S2: PCR mixture and PCR conditions for self-developed PCR assays; Table S3: Oligonucleotide primers used for sequencing of cloned amplicon, which overlapped the flavi-NS5-like protein gene and the fragment of host genome; Table S4: Data on *Ixodes ricinus* ticks that were PCR-positive on the Integrated Jingmenvirus polymerase gene; Table S5: Quantitative PCR assessment of the copy number of the inserted flavi-NS5-like protein gene in *I. ricinus* ticks; Table S6: The results of nested-PCR screening on JLV of other ixodid ticks (not *I. ricinus*).

## References

[1] Qin X.C., Shi M., Tian J.H., Lin X.D., Gao D.Y., He J.R., Wang J.B., Li C.X., Kang Y.J., Yu B. (2014). A tick-borne segmented RNA virus contains genome segments derived from unsegmented viral ancestors. Proc. Natl. Acad. Sci. USA.

[2] Wang Z.D., Wang B., Wei F., Han S.Z., Zhang L., Yang Z.T., Yan Y., Lv X.L., Li L., Wang S.C. (2019). A new segmented virus associated with human febrile illness in China. N. Engl. J. Med..

[3] Kholodilov I.S., Belova O.A., Morozkin E.S., Litov A.G., Ivannikova A.Y., Makenov M.T., Shchetinin A.M., Aibulatov S.V., Bazarova G.K., Bell-Sakyi L. (2021). Geographical and tick-dependent distribution of Flavi-like Alongshan and Yanggou tick viruses in Russia. Viruses.

[4] Kobayashi D., Kuwata R., Kimura T., Shimoda H., Fujita R., Faizah A.N., Kai I., Matsumura R., Kuroda Y., Watanabe S. (2021). Detection of Jingmenviruses in Japan with evidence of vertical transmission in ticks. Viruses.

[5] Jia N., Liu H.B., Ni X.B., Bell-Sakyi L., Zheng Y.C., Song J.L., Li J., Jiang B.G., Wang Q., Sun Y. (2019). Emergence of human infection with Jingmen tick virus in China: A retrospective study. EBioMedicine.

[6] Ternovoi V.A., Gladysheva A.V., Sementsova A.O., Zaykovskaya A.V., Volynkina A.S., Kotenev E.S., Agafonov A.P., Loktev V.B. (2020). Detection of the RNA for new multicomponent virus in patients with Crimean-Congo hemorrhagic fever in Southern Russia. Vestn. Ross. Akad. Meditsinskikh Nauk.

[7] Emmerich P., Jakupi X., von Possel R., Berisha L., Halili B., Günther S., Cadar D., Ahmeti S., Schmidt-Chanasit J. (2018). Viral metagenomics, genetic and evolutionary characteristics of Crimean-Congo hemorrhagic fever *Orthonairovirus* in humans, Kosovo. Infect. Genet. Evol..

[8] Kholodilov I.S., Litov A.G., Klimentov A.S., Belova O.A., Polienko A.E., Nikitin N.A., Shchetinin A.M., Ivannikova A.Y., Bell-Sakyi L., Yakovlev A.S. (2020). Isolation and Characterisation of Alongshan Virus in Russia. Viruses.

[9] Kuivanen S., Levanov L., Kareinen L., Sironen T., Jääskeläinen A.J., Plyusnin I., Zakham F., Emmerich P., Schmidt-Chanasit J., Hepojoki J. (2019). Detection of novel tick-borne pathogen, Alongshan virus, in *Ixodes ricinus* ticks, South-Eastern Finland, 2019. Eurosurveillance.

[10] Dinçer E., Hacıoglu S., Kar S., Emanet N., Brinkmann A., Nitsche A., Özkul A., Linton Y.-M., Ergünay K. (2019). Survey and characterization of Jingmen tick virus variants. Viruses.

[11] Guo J.-J., Lin X.-D., Chen Y.-M., Hao Z.-Y., Wang Z.-X., Yu Z.-M., Lu M., Li K., Qin X.-C., Wang W. (2020). Diversity and circulation of Jingmen tick virus in ticks and mammals. Virus Evol..

[12] Temmam S., Bigot T., Chrétien D., Gondard M., Pérot P., Pommelet V., Dufour E., Petres S., Devillers E., Hoem T. (2019). Insights into the host range, genetic diversity, and geographical distribution of Jingmenviruses. mSphere.

[13] Ternovoi V.A., Protopopova E.V., Shvalov A.N., Kartashov M.Y., Bayandin R.B., Tregubchak T.V., Yakovlev S.A., Nikiforov K.A., Konovalova Svetlana N., Loktev V.B. (2020). Complete coding genome sequence for a novel multicomponent kindia tick virus detected from ticks collected in Guinea. bioRxiv.

[14] Vandegrift K.J., Kumar A., Sharma H., Murthy S., Kramer L.D., Ostfeld R., Hudson P.J., Kapoor A. (2020). Presence of segmented *Flavivirus* Infections in North America. Emerg. Infect. Dis..

[15] Villa E.C., Maruyama S.R., de Miranda-Santos I.K.F., Palacios G., Ladner J.T. (2017). Complete coding genome sequence for Mogiana Tick Virus, a Jingmenvirus isolated from ticks in Brazil. Genome Announc..

[16] Yu Z.M., Chen J.T., Qin J., Guo J.J., Li K., Xu Q.Y., Wang W., Lu M., Qin X.C., Zhang Y.Z. (2020). Identification and characterization of Jingmen tick virus in rodents from Xinjiang, China. Infect. Genet. Evol..

[17] Maruyama S.R., Castro-Jorge L.A., Ribeiro C., Gardinassi L.G., Garcia G.R., Brandão L.G., Rodrigues A.R., Okada M.I., Abrão E.P., Ferreira B.R. (2014). Characterisation of divergent Flavivirus NS3 and NS5 protein sequences detected in *Rhipicephalus microplus* ticks from Brazil. Mem. Inst. Oswaldo Cruz.

[18] Gondard M., Temmam S., Devillers E., Pinarello V., Bigot T., Chrétien D., Aprelon R., Vayssier-Taussat M., Albina E., Eloit M. (2020). RNA viruses of *Amblyomma variegatum* and *Rhipicephalus microplus* and cattle susceptibility in the French Antilles. Viruses.

[19] Holmes E.C. (2011). The evolution of endogenous viral elements. Cell Host Microbe.

[20] Katzourakis A., Gifford R.J. (2010). Endogenous viral elements in animal genomes. PLoS Genet..

[21] Crochu S., Cook S., Attoui H., Charrel R.N., De Chesse R., Belhouchet M., Lemasson J.J., de Micco P., de Lamballerie X. (2004). Sequences of Flavivirus-related RNA viruses persist in DNA form integrated in the genome of *Aedes* spp. Mosquitoes. J. Gen. Virol..

[22] Roiz D., Vázquez A., Seco M.P.S., Tenorio A., Rizzoli A. (2009). Detection of novel insect Flavivirus sequences integrated in *Aedes albopictus* (Diptera: Culicidae) in Northern Italy. Virol. J..

[23] Suzuki Y., Frangeul L., Dickson L.B., Blanc H., Verdier Y., Vinh J., Lambrechts L., Saleh M.-C. (2017). Uncovering the repertoire of endogenous Flaviviral elements in *Aedes* mosquito genomes. J. Virol..

[24] Crava C.M., Varghese F.S., Pischedda E., Halbach R., Palatini U., Marconcini M., Gasmi L., Redmond S., Afrane Y., Ayala D. (2021). Population genomics in the arboviral vector *Aedes aegypti* reveals the genomic architecture and evolution of endogenous viral elements. Mol. Ecol..

[25] Spadar A., Phelan J.E., Benavente E.D., Campos M., Gomez L.F., Mohareb F., Clark T.G., Campino S. (2021). Flavivirus integrations in *Aedes aegypti* are limited and highly conserved across samples from different geographic regions unlike integrations in *Aedes albopictus*. Parasites Vectors.

[26] Lequime S., Lambrechts L. (2017). Discovery of Flavivirus-derived endogenous viral elements in *Anopheles* mosquito genomes supports the existence of *Anopheles*-associated insect-specific Flaviviruses. Virus Evol..

[27] Russo A.G., Kelly A.G., Tuipulotu D.E., Tanaka M.M., White P.A. (2019). Novel insights into endogenous RNA viral elements in *Ixodes scapularis* and other arbovirus vector genomes. Virus Evol..

[28] ter Horst A.M., Nigg J.C., Dekker F.M., Falk B.W. (2019). Endogenous viral elements are widespread in arthropod genomes and commonly give rise to PIWI-interacting RNAs. J. Virol..

[29] Filippova N.A. (1977). Ixodid Ticks of Subfamily Ixodinae // Fauna SSSR Paukoobraznye.

[30] Filippova N.A. (1997). Ixodid Ticks of Subfamily Ambyomminae // Fauna of Russia and Neighbouring Countries. Arachnoidea.

[31] Makenov M., Karan L., Shashina N., Akhmetshina M., Zhurenkova O., Kholodilov I., Karganova G., Smirnova N., Grigoreva Y., Yankovskaya Y. (2019). First detection of tick-borne encephalitis virus in *Ixodes ricinus* ticks and their rodent hosts in Moscow, Russia. Ticks Tick. Borne Dis..

[32] Kovalev S.Y., Golovljova I.V., Mukhacheva T.A. (2016). Natural hybridization between *Ixodes ricinus* and *Ixodes persulcatus* ticks evidenced by molecular genetics methods. Ticks Tick. Borne Dis..

[33] Scherer R. PropCIs: Various Confidence Interval Methods for Proportions. https://cran.r-project.org/package=PropCIs.

[34] Cowling D.W., Gardner I.A., Johnson W.O. (1999). Comparison of methods for estimation of individual-level prevalence based on pooled samples. Prev. Vet. Med..

[35] Sergeant E. Epitools Epidemiological Calculators. http://epitools.ausvet.com.au.

[36] Williams C.J., Moffitt C.M. (2001). A Critique of methods of sampling and reporting pathogens in populations of fish. J. Aquat. Anim. Health.

[37] Castresana J. (2000). Selection of conserved blocks from multiple alignments for their use in phylogenetic analysis. Mol. Biol. Evol..

[38] Kumar S., Stecher G., Li M., Knyaz C., Tamura K. (2018). MEGA X: Molecular evolutionary genetics analysis across computing platforms. Mol. Biol. Evol..

[39] Tavaré S. (1986). Some probabilistic and statistical problems in the analysis of dna sequences. Lectures on Mathematics in the Life Sciences.

[40] Tamura K. (1992). Estimation of the number of nucleotide substitutions when there are strong transition-transversion and G+C-content biases. Mol. Biol. Evol..

[41] Bolger A.M., Lohse M., Usadel B. (2014). Trimmomatic: A flexible trimmer for Illumina sequence data. Bioinformatics.

[42] Bankevich A., Nurk S., Antipov D., Gurevich A.A., Dvorkin M., Kulikov A.S., Lesin V.M., Nikolenko S.I., Pham S., Prjibelski A.D. (2012). SPAdes: A new genome assembly algorithm and its applications to single-cell sequencing. J. Comput. Biol..

[43] Altschul S.F., Gish W., Miller W., Myers E.W., Lipman D.J. (1990). Basic Local Alignment Search Tool. J. Mol. Biol..

[44] Stanojević M., Li K., Stamenković G., Ilić B., Paunović M., Pešić B., Maslovara I.Ð., Šiljić M., Ćirković V., Zhang Y. (2020). Depicting the RNA virome of hematophagous arthropods from Belgrade, Serbia. Viruses.

[45] Estrada-Peña A., Pfäffle M.P., Petney T.N. (2017). Genus *Ixodes* Latreille, 1795. Ticks of Europe and North Africa. A Guide to Species Identification.

[46] Bell-Sakyi L., Zweygarth E., Blouin E.F., Gould E.A., Jongejan F. (2007). Tick cell lines: Tools for tick and tick-borne disease research. Trends Parasitol..

[47] Charrier N.P., Hermouet A., Hervet C., Agoulon A., Barker S.C., Heylen D., Toty C., McCoy K.D., Plantard O., Rispe C. (2019). A Transcriptome-based phylogenetic study of hard ticks (Ixodidae). Sci. Rep..

[48] Ladner J.T., Wiley M.R., Beitzel B., Auguste A.J., Dupuis A.P., Lindquist M.E., Sibley S.D., Kota K.P., Fetterer D., Eastwood G. (2016). A Multicomponent animal virus isolated from mosquitoes. Cell Host Microbe.

[49] Aiewsakun P., Katzourakis A. (2015). Endogenous Viruses: Connecting recent and ancient viral evolution. Virology.

[50] Goic B., Stapleford K.A., Frangeul L., Doucet A.J., Gausson V., Blanc H., Schemmel-Jofre N., Cristofari G., Lambrechts L., Vignuzzi M. (2016). Virus-derived DNA drives mosquito vector tolerance to arboviral infection. Nat. Commun..

[51] Wallau G.L. (2022). RNA virus EVEs in insect genomes. Curr. Opin. Insect Sci..

[52] Tassetto M., Kunitomi M., Whitfield Z.J., Dolan P.T., Sánchez-Vargas I., Garcia-Knight M., Ribiero I., Chen T., Olson K.E., Andino R. (2019). Control of RNA viruses in mosquito cells through the acquisition of vDNA and endogenous viral elements. eLife.

[53] Petersen M., Armisén D., Gibbs R.A., Hering L., Khila A., Mayer G., Richards S., Niehuis O., Misof B. (2019). Correction to: Diversity and evolution of the transposable element repertoire in arthropods with particular reference to insects. BMC Ecol. Evol..

[54] Gilbert C., Peccoud J., Cordaux R. (2021). Transposable elements and the evolution of insects. Annu. Rev. Entomol..

[55] Whitfield Z.J., Dolan P.T., Kunitomi M., Tassetto M., Seetin M.G., Oh S., Heiner C., Paxinos E., Andino R. (2017). The diversity, structure, and function of heritable adaptive immunity sequences in the *Aedes aegypti* genome. Curr. Biol..

[56] Palatini U., Miesen P., Carballar-Lejarazu R., Ometto L., Rizzo E., Tu Z., van Rij R.P., Bonizzoni M. (2017). Comparative genomics shows that viral integrations are abundant and express pirnas in the arboviral vectors *Aedes aegypti* and *Aedes albopictus*. BMC Genom..

