# Supplementary material for: Integrated Jingmenvirus Polymerase Gene in Ixodes ricinus Genome
Source: Viruses. 2022 Aug 29;14(9):1908. doi: 10.3390/v14091908 (PMC9501327; doi:10.3390/v14091908)
Supplement: Supplementary file 1 [file viruses-14-01908-s001.zip › Table S2. PCR mixture and PCR conditions for self-developed PCR assays.pdf]

Table S2. PCR mixture and PCR conditions for self-developed PCR assays

Primers'/Assay's Name		PCR mixture	PCR conditions	
Nested PCR for screening on JMV				
Yanggou -OUT -F<br>JMTV-OUT-F<br>Alongshan-OUT -F<br>Yanggou-OUT -R<br>JMTV-OUT -R<br>Alongshan-OUT -R	Multiplex Mix Out	5 µL of cDNA or DNA<br>0.2 mM each dNTP<br>9 pmol of each primer<br>10 µl PCR buffer master mix (Amplisens, Moscow, Russia)<br><br>Total volume: 25 µl	95°C for 5 min<br>95°C for 10 s<br>52°C for 15 s<br>72°C for 15 s<br>72°C for 2 min	35 cycles
JMTV-IN -F<br>Alongshan-IN -F<br>Alongshan-IN-Rev<br>JMTV-IN -Rev<br>Yanggou-IN -Rev	Multiplex Mix In	1 µL of amplicons<br>0.2 mM each dNTP<br>9 pmol of each primer<br>10 µl PCR buffer master mix (Amplisens, Moscow, Russia)<br><br>Total volume: 25 µl	95°C for 5 min<br>95°C for 10 s<br>56°C for 15 s<br>72°C for 15 s<br>72°C for 2 min	35 cycles
PCR for sequencing of 509 bp fragment				
Mos-Seq-F-2380<br>Mos-Seq-R-2925		5 µL of cDNA or DNA<br>0.2 mM each dNTP<br>9 pmol of each primer<br>10 µl PCR buffer master mix (Amplisens, Moscow, Russia)<br><br>Total volume: 25 µl	95°C for 5 min<br>95°C for 10 s<br>64°C for 20 s<br>72°C for 20 s<br>95°C for 10 s<br>62°C for 20 s<br>72°C for 20 s<br>95°C for 10 s<br>60°C for 20 s<br>72°C for 20 s<br>72°C for 2 min	3 cycles<br>3 cycles<br>36 cycles
qPCR assay for the inserted Jingmenvirus polymerase gene and for Yanggou tick virus detection				
IRJV-NS5 -F<br>IRJV-NS5 -Rev<br>IRJV-NS5-Probe<br><br>IRJV-NS5 -F<br>YANGV-Rev<br>YANGV-Pr		10 µl DNA sample<br>0.2 mM each dNTP<br>9 pmol each primer<br>3 pmol fluorescent probe<br>0.5 µl TaqF DNA polymerase<br>5 µl PCR buffer (Amplisens, Moscow, Russia)<br><br>Total volume: 25 µl	95°C for 15 min<br>95°C for 10 s<br>55°C for 30 s	45 cycles
qPCR assay for the target site* detection				
Ir-3100-F<br>Ir-3100-R<br>Ir-3100-probe		10 µl DNA sample<br>0.2 mM each dNTP<br>9 pmol each primer<br>3 pmol fluorescent probe<br>0.5 µl TaqF DNA polymerase<br>5 µl PCR buffer (Amplisens, Moscow, Russia)<br><br>Total volume: 25 µl	95°C for 15 min<br>95°C for 10 s<br>55°C for 30 s	45 cycles
PCR for obtaining a long amplicon for cloning				
MS-F-875<br>MS-R-3100		20 µL volumes containing<br>10 µl DNA sample<br>9 pmol of each primer<br>0.3 µl Q5 High-Fidelity DNA Polymerase (New England Biolabs, M0491S)<br>5 µl Q5 Reaction Buffer<br><br>Total volume: 20 µl	98°C for 30 s<br>98°C for 10 s<br>58°C for 30 s<br>72°C for 75 s<br>72°C for 5 min	40 cycles
PCR for clones sequencing				

MS-F-875<br>MS-R-1810<br>MS-F-1710<br>MS-R-2410<br>Mos-Seq-F-2380<br>MosSeqTick-R-3100	5 µL of cDNA or DNA<br>0.2 mM each dNTP<br>9 pmol of each primer<br>10 µl PCR buffer master mix (Amplisens, Moscow, Russia)<br><br>Total volume: 25 µl	95°C for 5 min<br>95°C for 10 s<br>58°C for 20 s 42 cycles<br>72°C for 20 s<br>72°C for 2 min
