# Supplementary material for: Integrated Jingmenvirus Polymerase Gene in Ixodes ricinus Genome
Source: Viruses. 2022 Aug 29;14(9):1908. doi: 10.3390/v14091908 (PMC9501327; doi:10.3390/v14091908)
Supplement: Supplementary file 1 [file viruses-14-01908-s001.zip › Table S3. Oligonucleotide primers used for sequencing of cloned amplicon.pdf]

Table S3. Oligonucleotide primers used for sequencing of cloned amplicon, which overlapped the flavi-NS5-like protein gene and the fragment of host genome

Name	Nucleotide sequence (5'–3')
MS-F-875	TCGGATAGGCTGGAGACTCA
MS-R-1810	TGGAGTGTCGCAGGACAGGGA
MS-F-1710	ATCCAGTATCTTCCAGCGGA
MS-R-2410	TGGCCTGTGAAGTACGGTGAG
Mos-Seq-F-2380	TGCTCCCATCAGTACTGGCCT
MosSeqTick-R-3100	TCGGGAACCGGCATCTCGTCA
