# Supplementary material for: Integrated Jingmenvirus Polymerase Gene in Ixodes ricinus Genome
Source: Viruses. 2022 Aug 29;14(9):1908. doi: 10.3390/v14091908 (PMC9501327; doi:10.3390/v14091908)
Supplement: Supplementary file 1 [file viruses-14-01908-s001.zip › Table S5. Quantitative PCR assessment of the copy number of the inserted flavi-NS5-like protein gene in I. ricinus ticks.pdf]

Table S5. Quantitative PCR assessment of the copy number of the inserted flavi-NS5-like protein gene in *I. ricinus* ticks

Sample ID	Sex/ Stage	Blood feeding status	Copy number per sample		Number of cells per sample	Copy number of inserted flavi-NS5-like protein gene, per cell
			Inserted flavi-NS5-like protein gene	ITS2		
Mos49	Nymph	Unfed	156,320	163,839,047	172,462	0.9
Mos61	Nymph	Unfed	216,662	144,964,867	152,595	1.4
Mos67	Nymph	Unfed	169,007	67,708,411	71,272	2.4
Mos73	Nymph	Unfed	399,896	255,257,998	268,693	1.5
Mos80	Nymph	Unfed	101,000	95,447,258	100,471	1.0
Mos84	Female	Unfed	2,563,541	312,694,862	329,153	7.8*
Mos85	Female	Unfed	712,428	129,626,877	136,449	5.2*
Mos102	Male	Unfed	1,490,792	444,466,657	467,860	3.2
Mos193	Nymph	Unfed	84,936	61,276,147	64,501	1.3
Mos213	Nymph	Unfed	164,318	116,248,275	122,367	1.3
Mos194	Nymph	Unfed	135,626	186,401,821	196,212	0.7
Mos219	Male	Unfed	494,721	160,680,448	169,137	2.9
Mos223	Male	Unfed	181,443	75,002,040	78,950	2.3
Mos224	Male	Unfed	368,983	130,759,288	137,641	2.7
Mos228	Male	Unfed	311,733	258,075,150	271,658	1.1
Mos231	Female	Unfed	520,949	634,652,965	668,056	0.8
Mos723	Female	Partially fed	2,830,093	1,906,771,278	2,007,128	1.4
Mos727	Female	Partially fed	1,974,353	1,115,201,924	1,173,897	1.7
					Median	1.4

* - these two values were not used in the median calculation as outliers exceeding 1.5*IQR
