# Supplementary material for: Integrated Jingmenvirus Polymerase Gene in Ixodes ricinus Genome
Source: Viruses. 2022 Aug 29;14(9):1908. doi: 10.3390/v14091908 (PMC9501327; doi:10.3390/v14091908)
Supplement: Supplementary file 1 [file viruses-14-01908-s001.zip › Table S6. The results of nested-PCR screening on JLV of other ixodid ticks.pdf]

Table S6. The results of nested-PCR screening on JLV of other ixodid ticks (not *I. ricinus*)

Species	Region	Date of tick collection	Number of ticks	Number of formed pools	Number of PCR+ on JLV pools	Identification of positive samples
Ixodes persulcatus	Republic of Karelia	2013	25	7	1	Alongshan virus
Ixodes persulcatus	Republic of Karelia	2014	10	3	0	
Ixodes persulcatus	Republic of Karelia	2015	2	2	0	
Ixodes persulcatus	Kemerovo region	2014	5	5	0	
Ixodes persulcatus	Republic of Altai	2016	78	78	0	
Ixodes trianguliceps	Moscow region	2017	12	2	0	
Ixodes trianguliceps	Moscow region	2019	3	3	0	
Dermacentor reticulatus	Republic of Altai	2016	53	53	0	
Dermacentor marginatus	Republic of Altai	2016	25	25	2	Yanggou tick virus
Dermacentor silvarum	Republic of Altai	2016	119	119	0	
Dermacentor nuttalli	Republic of Altai	2016	45	45	2	Alongshan virus
Haemaphysalis concinna	Republic of Altai	2016	8	8	0	
Haemaphysalis concinna	Altai Territory	2016	17	17	2	Alongshan virus
Haemaphysalis japonica	Khabarovsk Territory	2016	10	10	0	
